# Comprehensive Analysis of MicroRNA–Messenger RNA from White Yak Testis Reveals the Differentially Expressed Molecules Involved in Development and Reproduction

**DOI:** 10.3390/ijms19103083

**Published:** 2018-10-09

**Authors:** Quanwei Zhang, Qi Wang, Yong Zhang, Shuru Cheng, Junjie Hu, Youji Ma, Xingxu Zhao

**Affiliations:** 1 College of Veterinary Medicine, Gansu Agriculture University, Lanzhou 730070, China; zhangqw@gsau.edu.cn (Q.Z.); forever45214125@126.com (Q.W.); Hujj@gsau.edu.cn (J.H.); 2 College of Life science and Technology, Gansu Agriculture University, Lanzhou 730070, China; 3 College of Animal Science and Technology, Gansu Agriculture University, Lanzhou 730070, China; chengsr@gsau.edu.cn (S.C.); mayj@gsau.edu.cn (Y.M.)

**Keywords:** microRNA–messenger RNA, development, reproduction, spermatogenesis, yak

## Abstract

Testis development is a vital and tightly regulated process in mammals. Understanding the biological mechanisms underlying testis development will benefit the animal reproduction industry. Expression changes in microRNA and messenger RNA in response to dynamic regulation effects have been associated with this process. However, very little is known about the roles of these molecules in yak development. Using whole-genome small RNA and messenger RNA sequencing, we performed a comprehensive analysis of the microRNA–messenger RNA interaction network expression in the testicles of Tianzhu white yaks during three developmental stages. Using Short Time-series Expression Miner analysis we identified 589 differentially expressed microRNAs (DERs) and 3383 differentially expressed messenger RNAs (DEGs) in the three age groups. A total of 93 unique DEGs are primarily involved in reproduction and testis development. Subsequently, four integration networks were constructed according to the DEGs and DERs in three biological processes. Nineteen DEGs were potentially regulated by 60 DERs, of which miR-574 and target gene *AURKA* played a crucial role in yak testis development and reproduction. The results of this study provide a basis for further exploration of the microRNA–messenger RNA interactions in testis development and reproduction and aid in uncovering the molecular mechanisms of spermatogenesis in male mammals.

## 1. Introduction

Tianzhu white yak (*Bos grunniens*) is one of the yak breeds, and it is a unique and rare domesticated animal in Tianzhu Tibetan autonomous county (Wuwei City, Gansu Province, China). The Tianzhu white yak population is no more than 100,000, comprising 0.7% of the yak global population [1], and this low breeding population is associated with poor production performance [2]. Yaks have been grazed for a long period without supplementary feeding [3], which has led to poor nutrition, delay of sexual maturation, and reproductive performance degradation [4]. Therefore, it is of practical significance to understand and to master the reproductive and physiological characteristics of yaks. However, very little is known about molecular roles in the development and reproduction of Tianzhu white yaks, especially in yak testis.

The main function of the testis is to produce sperm and synthesize hormones. Testis development can be divided into the fetal, infantile/juvenile, and puberty stages [5]. During these stages, testis development involves mainly localization and differentiation of sertoli cells, primordial germ cells, and spermatogonium, comprising a series of highly regulated molecular and cellular processes and drastic morphological changes. Spermatogenesis is a strictly regulated processes, requiring precise control of a large number of genes and networks acting synergistically or antagonistically at the transcriptional and post-transcriptional levels [6]. Therefore, identifying the regulatory principles that govern testis development is of great interest to biologists studying animal molecular breeding. In particular, microRNAs already have been studied widely in organ development because of their inhibitory effects on target genes.

MicroRNAs are small noncoding RNAs that participate in numerous biological processes [7]. MicroRNAs have been increasingly recognized as important participants that regulate cellular processes by complementary binding to target messenger RNA. The regulation of microRNAs occurs in the developmental stage and in a tissue-specific fashion [8]. The microRNA and messenger RNA interaction networks likely regulate most biological processes and can be employed to understand complex processes, such as genetic variation and specific diseases. Recently, multiple studies have demonstrated the importance of microRNAs in many biological processes with an integrative approach in animals [9,10]. Moreover, numerous studies have sequenced and analyzed microRNA or transcriptome profiles with various approaches in different species and tissues. These studies have suggested that the expression of microRNAs has a fixed pattern in animals. However, this fixed pattern has differences among the various species and tissues.

Recent evidence has suggested that the differences were mainly expression in kinds and quantity, particularly in the testis. It has been reported that there were 112 specific microRNAs expressed in human testis [11]. However, 49 microRNAs were identified in adult mice [12] and 21 microRNAs in cattle testicles [13]. Numerous microRNAs had been reported to be associated with testis development and spermatogenesis. For example, miR-19, miR-221/222, miR-122, and miR-888 have been demonstrated to play essential roles in testis development and spermatogenesis [14]. However, much of the information related to development remains incomplete, in particular that related to the reproduction and development of the yak testis. Understanding the regulatory roles of microRNAs in yak testicular development and reproduction not only complements the theoretical basis for the study of reproductive diseases in yak breeding, but also provides new ideas and approaches for improving the semen quality and breeding capacity in animal husbandry.

Therefore, in the present study, the expression patterns of microRNA–messenger RNA were analyzed in yak testis during different developmental stages. This study can elucidate potentially dysregulated microRNAs/messenger RNAs and clarify the possible roles of the critical proteins in signaling pathways enriched by the differential target genes of the dysregulated microRNAs. These data allow researchers to better understand the potential mechanisms of testis development and reproduction, contributing to the genetic breeding knowledge base and improving reproductive performance of domestic livestock.

## 2. Results

### 2.1. Identifying Differentially Expressed Genes of Transcriptome Sequencing

**Figure 1:**
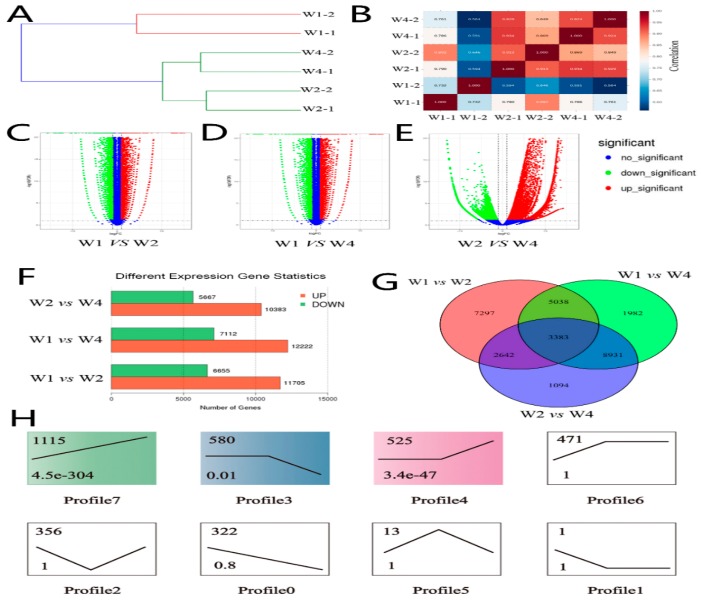
Identifying differentially expressed genes by transcriptome sequencing. (**A**) The hierarchical clustering was carried out for intergroup analysis correlation. (**B**) Sample correlation heat-map was performed according to the expression profiles for quality control. (**C**–**E**) The volcano plot of significant differentially expressed genes (DEGs) in three groups (W1, W2, and W3). Blue represents no significant difference. Green represents significantly downregulated DEGs. Red represents significantly upregulated DEGs. A total of 16,050, 19,334, and 18,306 DEGs were identified with pairwise alignment in intergroup. (**F**) The statistical graph of significant intergroup DEGs. Green represents downregulated genes. Red represents upregulated genes. (**G**) Venn diagram of differentially expressed genes in three groups. (**H**) Profile analysis of 3383 DEGs by STEM.

The average clean data of transcriptome sequencing was 524,399,092 bp per samples and the high quality rate was not lower than 98% in each sample (the detail characteristics of NGS sequencing showed in Supplement File Table S1). Stringent quality control was carried out based on a previously reported method [15]. The clean data of the transcriptome were used for predicting the differentially expressed genes (DEGs) by multiple comparisons in three groups (Figure 1). Hierarchical clustering (Figure 1A) was performed and the sample correlation heat-map (Figure 1B) was constructed according to the expression profiles for quality control. All samples in the different groups were divided into two clusters. The results showed that the 2-year-old yak may be the first stage used for mating and reproduction. A total of 16,050, 19,334, and 18,306 DEGs were identified by pair-wise alignment between groups W1 vs. W2, W1 vs. W4, and W2 vs. W4, respectively (Figure 1C–E). Among these, 64.7%, 63.2%, and 63.9% DEGs were upregulated, respectively (*FDR* < 0.05 and *log2FC >* 1). The number of upregulated DEGs was approximately 1.7 times greater than the downregulated genes (Figure 1F). Transcriptomic analysis revealed that 62.8 to 75.6% of genes of the yak genome were differentially expressed in testis samples in the different stages. A Venn diagram was constructed with DEGs after overlapping the repeats in three groups. We found that 8421, 6025, and 12,296 DEGs were co-expressed by pair-wise alignment groups, which had an increased intergroup tendency with the development of yak testis. We found 3383 DEGs were co-expressed in three stages (Figure 1G and Supplemental File Table S2) by multiple comparisons. Profile analysis was performed, based on 3383 DEGs, by Short Time-series Expression Miner (STEM) software (Figure 1H). As a result, eight profiles were identified, three of which (profiles 7, 4, and 3), including 2220 DEGs, were significantly different (*p* < 0.05). The remaining five profiles (profiles 0, 1, 2, 5, and 6) included 1163 DEGs, but exhibited no significant difference (*p* > 0.05).

### 2.2. Identifying Differentially Expressed MicroRNA of Small RNA Sequencing

**Figure 2:**
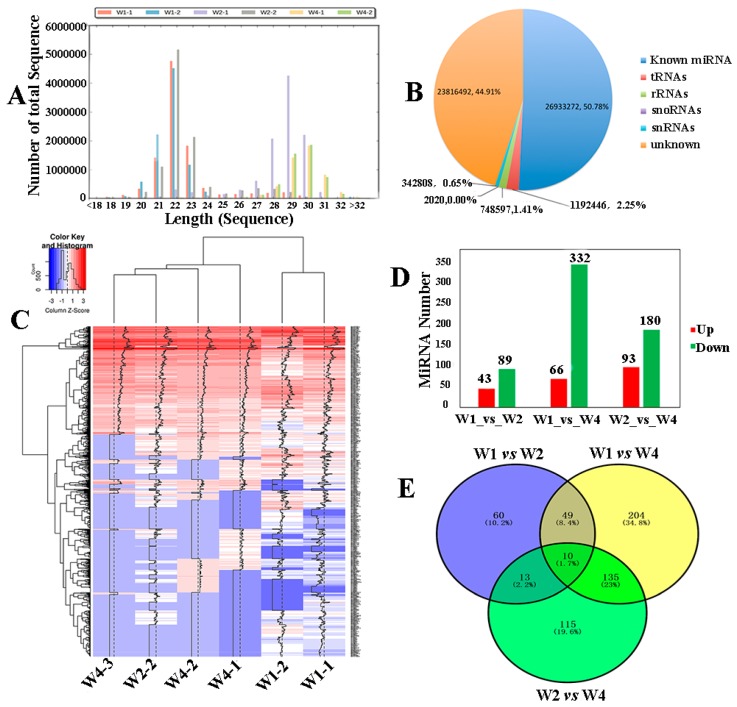
Identifying differentially expressed microRNA of small RNA sequencing. (**A**) The fragment length distribution of microRNAs in Tianzhu white yak testis. (**B**) The percentage statistics of small RNA in all samples. (**C**) The heatmap of all microRNAs in the three groups. (**D**) The statistical graph of the differentially expressed miRNAs (DERs) between groups. (**E**) The Venn diagram was constructed based on the aforementioned DERs by multiple comparisons of the three groups.

For small RNA sequencing, the number of unique reads was 10,672,605 bp per sample and the match rate (matching the small RNA databases) was not lower than 90% in each sample (Supplemental File Table S3). The unique reads of small RNA sequencing were used for predicting the microRNA by mirdeep2 software according to microRNA homology and high conservation (Figure 2). We first analyzed the fragment length of microRNAs from the Tianzhu white yak testis (Figure 2A) and found two peaks (≈22 bp and >28 bp) in the microRNAs length distribution. In the infant/juvenile ages (W1 to W2), 45% of the small RNA detected were microRNAs with a length of ≈22 bp. In the later developmental stage, the fragment length of majority small RNAs gradually increased to >28 bp in length, suggesting a dynamic change where microRNAs were replaced by PIWI-interacting RNAs (piRNAs are a class of newly discovered small, noncoding RNAs that are approximately 24 to 32 nucleotides in length) as the highest concentration detected. The types and percentages of small RNA were further identified and statistics of all samples are shown in Figure 2B. In the miRbase database (release 21), a total of 50.78% of the microRNAs were known, while 44.91% microRNA were unknown. Of the existing microRNAs, known microRNAs and predicted microRNAs were overlapped to obtain expression profiles (Figure 2C), 1832 microRNAs (77.8% known microRNA and 22.2% novel microRNA) and 14,518 potential target genes were identified (Supplemental File Table S4). After applying stringent filtering criterion (fold change> 2 and *P < 0.05*) by pairwise intergroup alignment, 803 microRNAs were identified, 25.2% of which were upregulated and 74.8% downregulated. Multiple intergroup comparisons were applied to identify the differentially expressed microRNAs (DERs), and we found 132, 273 and 398 significantly DERs in the three groups (Figure 2D). After overlapping the repeated microRNAs, 589 unique DERs were found (Figure 2E). Most of the DERs were differentially expressed during testis development, but we found that there are ten co-expressed DERs including six known DERs gradually downregulated and four DERs gradually upregulated with increasing age. 

### 2.3. Gene Ontology (GO) and Pathways Analysis of Different Expressed Target Genes

**Figure 3:**
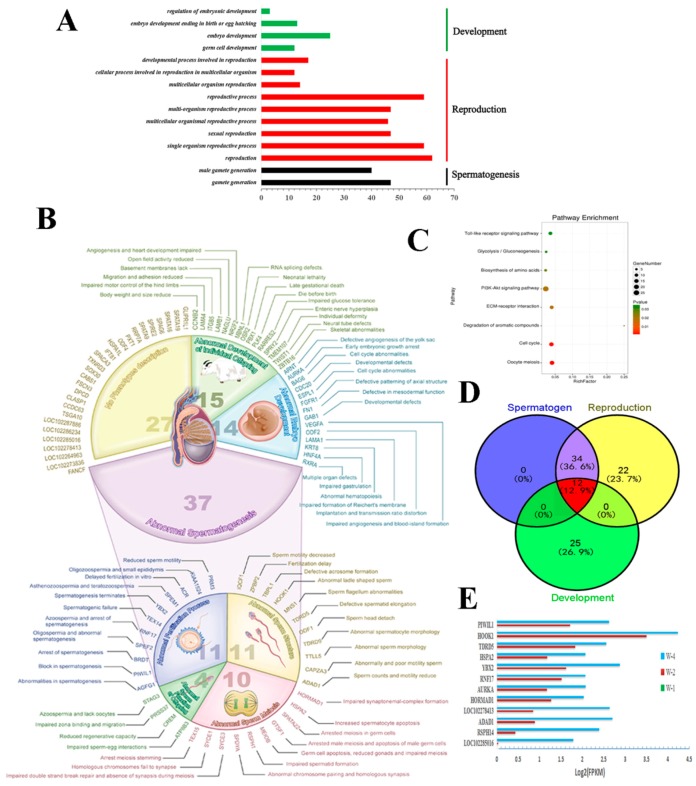
Gene ontology (GO) analysis of different expressed genes in transcriptome and small RNA sequencing. (**A**) The GO terms were clustered into three biological processes (development, reproduction, and spermatogenesis). (**B**) The potential function prediction and annotation of 93 unique DE genes from the MGI database based on homology. (**C**) Pathway enrichment of three significantly different profiles. (**D**) The Venn diagram was constructed based on 93 DEGs. (**E**) The relative expression levels of identified DEGs were calculated by the FPKM value.

The predicted target genes of DERs and transcriptome DEGs were overlapped for GO (Gene Ontology) and pathway analysis. A total of 2220 significantly DEGs in the three groups were mapped to the GO database for enrichment analysis (Figure 3). We identified 25, 12, and 30 significantly different GO terms (*p* < 0.05 and *Q* < 0.05) participating in biological process, cellular component and molecular function, respectively (Supplemental File Figure S1). Most of the GO terms had close relationships with testis development and reproduction. The GO terms were clustered into three biological processes (development, reproduction, and spermatogenesis) (Figure 3A). A total of 93 unique DEGs were found in these three processes after overlapping the repeat genes in similar GO terms. These genes were input into the Mouse Genome Informatics (MGI) database for predicting possible phenotypes or function based on the homology of genes (Supplement File 2 Table S5). The results revealed that 71% of these DEGs were annotated; possible phenotypes were predicted after knockout in a laboratory mouse model [16], and only a few genes, such as *STAG3* [17], *TDRD5* [18], and *RNF17* [19], were demonstrated to have biological functions associated with reproduction and gametogenesis. We found that mutations in most of these genes in model mice caused homozygous mice to display a knockout allele which exhibited male infertility because of a series abnormal generative processes (Figure 3B). However, 29% of these DEGs were not annotated with possible phenotypes, such as *SOX30* and *SPAG6*, that have been reported to play important roles in testis development [20,21]. For pathway analysis, we screened eight significant pathways (*p* < 0.05 and *Q* < 0.05), particularly cell cycle and oocyte meiosis pathways (Figure 3C). A Venn diagram was constructed based on these DEGs in development, reproduction, and spermatogenesis processes, and a total of 12 DEGs were co-expressed in these processes (Figure 3D). Notably, the expression level of four DEGs (*HSPA2*, *PIWIL1*, *TDRD5*, and *RNF17*) in the adult phase (W4) were 100 times higher than that in the pubescent phase (W2). The Fragments Per Kilobase of transcript per Million mapped reads (FPKM) value of these 12 DEGs gradually increased. The relative expression levels of these DEGs were calculated by the FPKM value (Figure 3E). Interestingly, all of the twelve DEGs were upregulated within the development stage and unexpressed in the early development stage of yak testis. We found 10 co-expressed microRNAs and 12 co-expressed DEGs in the three development ages that no single microRNA interacted with to regulate these target genes.

### 2.4. RT-PCR and Western Blot Validation of DERs and DEGs

**Figure 4:**
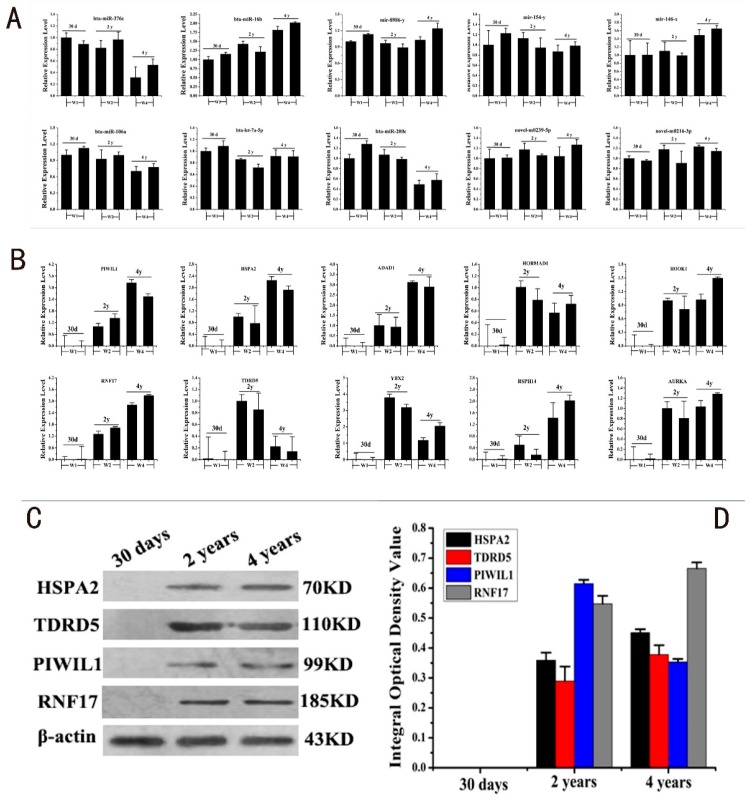
RT-PCR and Western blot assays for validating DERs and DEGs. (**A**) Stem-loop PCR assay for ten differentially co-expressed DERs. (**B**) Real-time PCR assay for ten differentially co-expressed DEGs. (**C**) Western blot assays for four highly co-expressed proteins in the three developmental stages. (**D**) The statistical graph for the four highly co-expressed proteins.

Stem-loop Polymerase Chain Reaction (PCR), real-time PCR, and Western blot were performed to validate the results of the 10 co-expressed DERs, DEGs, and target proteins (Figure 4). The Stem-loop PCR results of DERs suggested that all DERs were differentially expressed in the three development stages. Four DERs were significantly downregulated, four DERs were significantly upregulated, and two DERs were not remarkably regulated at the expression level (Figure 4A). This trend was in accordance with the predicted results of small RNA sequencing. The real-time PCR results of 10 DEGs were upregulated within the developmental stage and unexpressed in the early development stage of yak testis, suggesting that the 10 co-expressed DEGs were not detected in the early development stage. The relative expression levels of six of the DEGs were upregulated while three of the DEGs were downregulated from the pubertal to adult period in yak testis. The relative expression level of *AURKA* was upregulated, but not obviously (Figure 4B). We choose four target proteins, HSPA2, PIWIL1, TDRD5, and RNF17, with the highest expression levels in the adult phase for Western blot assays (Figure 4C). Target proteins were differentially expressed in two developmental ages, but were undetected in samples of 30 day old yaks; the expression tendency and expression levels were similar to that of the target genes (Figure 4D).

### 2.5. Integrated Network Analysis of Differentially Expressed MicroRNA–Messenger RNA in Testis Development, Reproduction and Spermatogenesis

**Figure 5:**
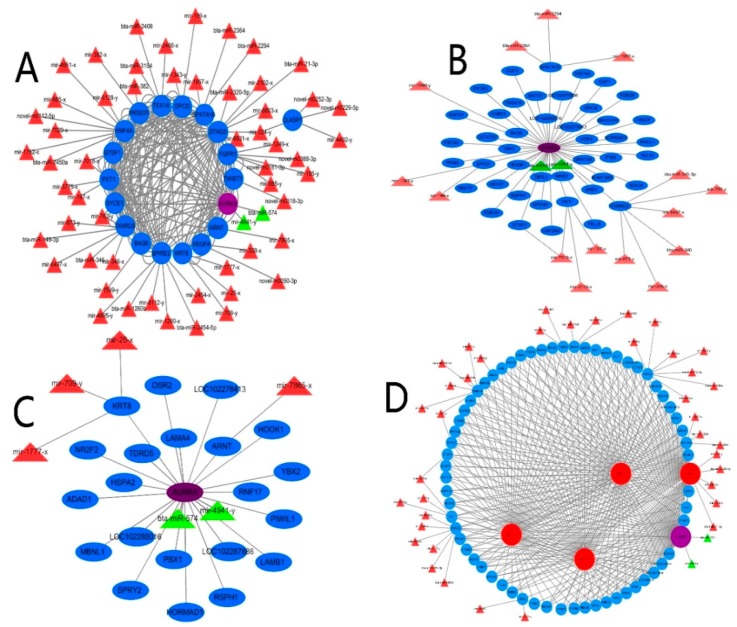
Integrated network analysis of different expressed microRNAs /messenger RNA. (**A**) The paired microRNA–messenger RNA interaction relationships of 93 DEGs and 589 DE microRNAs were used for creating an integrated network. (**B**) Only three in 38 candidate DEGs were potentially regulated by six microRNAs (*ρ* > 0.8) in testis development. Twenty-one DEGs have a close relationship compared with AURKA (*r* > 0.9). (**C**) In spermatogenesis, we identified seven out of 46 candidate DEGs that were potentially regulated by 17 microRNAs (*ρ* > 0.8). (**D**) There were 13 out of 68 candidate DEGs that were potentially regulated by 40 microRNAs (*ρ* > 0.8) in reproduction. Sixty-six DEGs had a close relationship, compared with AURKA (*r* > 0.9).

The paired microRNA–messenger RNA interaction relationship of 93 DEGs and 589 DERs was used for constructing an integrated network (Figure 5). Nineteen (20.4%) of the 93 DEGs were potentially regulated by 60 DERs. However, 79.6% of the candidate DEGs we considered particularly interesting in testis development, reproduction, and spermatogenesis were not found to have potential target microRNAs (Figure 5A). More interestingly, *AURKA* was the only gene in the 10 co-expressed DEGs that was regulated by bta-miR-574 and mir-4941-y; the Pearson value of AURKA was greater than 0.8, compared with other candidate DEGs. Therefore, AURKA was taken as the core DEG for the construction of the interaction network of microRNA–messenger RNA and DEGs in testis development, spermatogenesis, and reproduction. In testis development, we identified 38 candidate DEGs. A total of three DEGs were potentially regulated by six DERs (*ρ* > 0.8), indicating negative correlation. The Pearson correlation coefficient of 21 DEGs was greater than 0.9 compared with *AURKA* (*r* > 0.9), specifically *HSPA2*, *TDRD5*, and *PIWIL1* (r > 0.99), indicating that these genes were highly relevant and strongly interacting (Figure 5B). In spermatogenesis, we identified 46 candidate DEGs; seven were potentially regulated by 17 microRNAs (*ρ* > 0.8). The Pearson correlation coefficient of 44 DEGs was greater than 0.99, compared with *AURKA* Figure 5C). In reproduction, we identified 68 candidate DEGs, 13 of which were potentially regulated by 40 microRNAs (*ρ* > 0.8). For these DEGs, the Pearson correlation coefficient of 26 was larger than 0.99 (Figure 5D). These results suggested that AURKA and, potentially microRNAs, have crucial roles in these processes.

### 2.6. The Verification Analysis of Core DERs and DEGs of Yak Testis in Development and Reproduction

**Figure 6:**
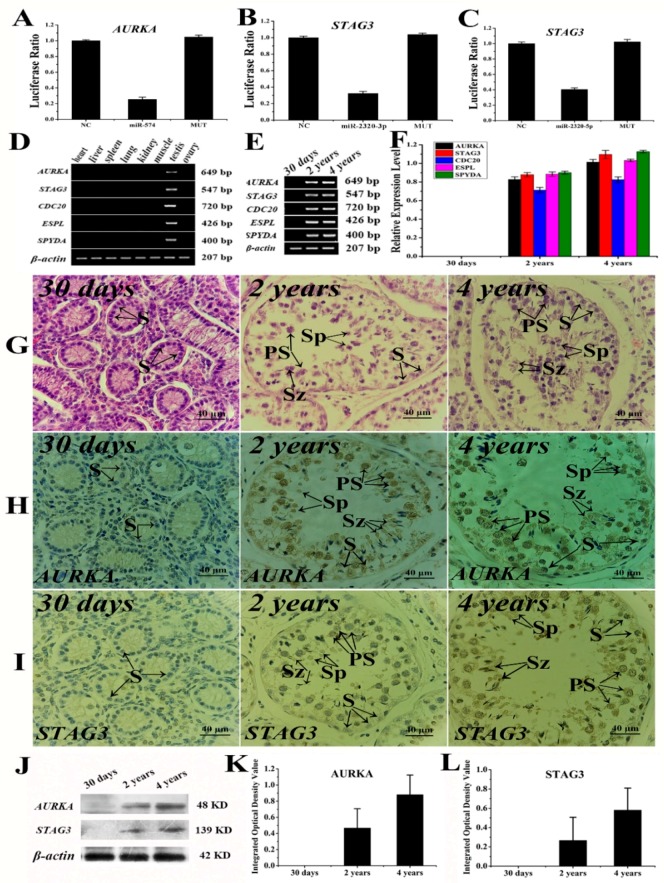
The crucial role of AURKA of yak testis in development and gametogenesis. (**A**) Validating *AURKA* as a target of miR-574 using a dual luciferase reporter assay. (**B**,**C**) Validating STAG3 as a target of miR-21-3p and miR-2320-3p using a dual luciferase reporter assay. (**D**) The crucial DEGs of oocyte meiosis were chosen for evaluating the role of *AURKA* in different tissues by RT-PCR. (**E**) The crucial DEGs were detected in testis samples of different development stages by RT-PCR. (**F**) The relative expression level of these DEGs was gradually upregulated with testis development. (**G**) H&E staining of testis samples at different developmental stages. (**H**,**I**) Immunohistochemical stain assay for expression and location of AURKA and STAG3 in testis samples in the different development stages. (**J**) Western blot assay for detecting the expression level of AURKA and STAG3 in testis samples in different developmental stages. (**K**,**L**) Relative protein expression level of AURKA and STAG3.

According to the microRNA–messenger RNA interaction analysis, a series of assays was carried out for validating in yak testis (Figure 6). We chose three microRNAs (miR-574, miR-21-3p, and miR-2320-5p) and target genes (*ARUKA* and *STAG3*) for assessing the validity of microRNA–messenger RNA interactions using dual luciferase assays. The luciferase activity of wild-type *AURKA* reporter cotransfected with miR-574 mimics was decreased by 25.4%, compared to the negative control (Figure 6A). The luciferase activity of wild-type *STAG3* reporter cotransfected with miR-21-3p and miR-2320-3p mimics was decreased by 32.4% (Figure 6B) and 40.4% (Figure 6C), respectively, compared to the negative control. The crucial DEGs of the oocyte meiosis pathway with significant differences were chosen for evaluating the role of *AURKA* in gametogenesis using RT-PCR. We found that DEGs were expressed only in testis (Figure 6D). The relative expression levels of these DEGs were differed among the different age groups (Figure 6E), and it was not detected in 30-day old testis samples. The relative expression levels of these DEGs were gradually upregulated with the advance of testis development (Figure 6F). Subsequently, the testis samples from the different stages were used for expression location. The spermatogonia were only observed in the early stage. The seminiferous tubules and germ cells displayed gradual increases in proliferation and differentiation with testis development, and were obviously observed in later stages (Figure 6G). AURKA and STAG3 were not expressed in 30-day old yak testis tissues (Figure 6H,I). AURKA was expressed particularly in the sperm head of 2-year old yak testis. However, AURKA was highly expressed in primary and secondary spermatocytes of 4-year old yak testis. STAG3 was expressed and located in all germ cells, but especially in spermatids and primary spermatocytes of 4-year old yak testis. The target proteins of AURKA and STAG3 were detected by Western blot (Figure 6J), but remained undetected in 30-day old testis tissues. The protein expression level of AURKA and STAG3 was gradually upregulated with testicular growth and development and the expression level of AURKA was twice that of STAG3 (Figure 6K,L).

## 3. Discussion

Development of the reproductive organs is a major factor that affects mammalian reproduction. It is driven by distinct programs of gene regulation and cellular organization [22]. Previous studies have established functions for specific microRNAs in regulating reproduction [23]. However, only a few microRNAs and target genes involved in the development and reproduction of testis have been identified in mammals [23,24,25]. Recently, it has been reported that a total of 61 DERs and 80 DEGs have been identified between cattleyak (cattle^♂^ and yak^♀^) and yak testes [26]. These results may be more beneficial to the understanding of male infertility mechanisms in cattleyak. However, there may be undiscovered DERs and DEGs that play important roles in yak testicular development and reproduction

In the present study, a comprehensive analysis of microRNA–messenger RNA was carried out in yak testis samples from the three developmental stages. Due to the smaller populations and lower birth and survival rates of yaks [27,28], there were only two available samples in each stage during this study. Stringent quality control was carried out, and the results show that two years of age may be a demarcation point in reproduction. Pastoralists often mate male yaks starting from the ages of 2 to 3 years and the pregnancy percentage of female yaks was up to 91%. Subsequently, transcriptome analysis revealed that 62.8–75.6% of genes in the yak genome were differentially expressed in the different developmental stages of yak testes. This finding suggests that testis development plays a dynamic regulatory role in gene expression. In order to obtain some pivotal candidate genes, DEGs were identified by STEM and multiple comparison analysis. A total of 3383 DEGs clustered into eight expression patterns, of which three profiles, including 2280 DEGs, were significantly different. Small RNA sequencing was performed and we found that microRNA levels show dynamic variation during testis development. The quantity of microRNA gradually decreased while the fragment length increased, evolving into piRNAs that are essential for reproduction [29]. However, studies of piRNAs in sex gland tissues are limited, and the mechanism needs to be elucidated in further studies. According to the biological function of testis [30], we found 93 DEGs associated with reproduction, gametogenesis, and testicle development. For example, *HSPA2* has been established as a measure of human sperm cellular maturity and fertility [31]. Seventy-one percent of these DEGs were annotated and predicted the possible phenotypes by MGI database [16]; only a few genes have been associated with reproduction and gametogenesis, such as *STAG3* [17], *TDRD5* [18], and *RNF17* [19]. However, 29% of these DEGs were not annotated, such as *SOX30* and *SPAG6*, which have been reported to play important roles in testis development [20,21]. This suggests that further studies are required in order to elucidate the roles of these genes.

Pathway analysis revealed that the DEGs of the oocyte meiosis pathway were specifically expressed in testis. However, most DEGs of the cell cycle pathway did not belong to 93 DEGs, except *Cdc20* and *ESPL1*. It has been reported that *Cdc20* plays a crucial role in reproduction [32,33]. Interestingly, we found 10 co-expressed microRNAs and 12 co-expressed DEGs in the three stages that did not interact with any of the target genes. For example, miR-200c [34], let-7a-3p [35], and miR-106a [36] have been reported to be associated with testis development and reproduction in mice. However, the target genes potentially regulated by these microRNAs were not in accordance with our results, suggesting that the regulation mechanism in male yak reproduction is not equivalent to that in mice. The results of integration networks revealed most of the DEGs and DERs have no interactions in yak testis development and reproduction. Interestingly, *AURKA* was the only gene in 10 co-expressed genes that have close interactions with *HSPA2*, *TDRD5*, *STAG3*, and *PIWIL1*, which are important functional genes for animal reproduction [17,18,31,37]. In addition, we found that *STAG3* is the executive functional gene for proper pairing and segregation of chromosomes during meiosis [17]. The results of verification assays demonstrated that *AURKA* and *STAG3* were differentially expressed in yak testis after two years of age. The subcellular localization of AURKA and STAG3 suggested that the highest expression was presented in primary spermatocytes, in which the major variations include homologous chromosome synapsis, spindle formation, and sister chromatid separation [38,39]. However, an important future study will be to reveal the role of these DEGs in reproduction because animal development and reproduction is a complicated and multigene regulated process, of which our understanding remains incomplete. Herein, we analyzed the microRNA–messenger RNA interaction relationship in yak testis so as to uncover potential deregulation of microRNAs/messenger RNA and expound the possible roles of the critical proteins in signaling pathways enriched by the differential target genes of the deregulated microRNA. This was done to better understand the potential mechanism of testis development and spermatogenesis, our paper contributes to genetic breeding and improving the reproductive performance of domestic yaks and other mammals.

## 4. Materials and Methods

### 4.1. Samples Preparation and Collection

Domesticated male white yaks (same male parent and different female parent, *n* = 6) from Tianzhu City (Gansu province, China) of different ages were selected for this study. All calves had been grazed in high-altitude pastures. Their birthdate (in March 2012) and body weight were recorded. The infantile samples consisted of 2 neonatal yaks (W1 group, age 30 days after birth, average body weight 6.5 ± 1.2 kg, W1-1, and W1-2). The pubescent samples included 2 sexually mature yaks (W2 group, age: 2 years old, average body weight: 157.61 ± 2.54 kg, W2-1, and W2-2). The 2 adult animals were mature yaks (W4 group, age: 4 years old, average body weight: 270.62 ± 3.68 kg, W4-1 and W4-2). The testis samples were obtained immediately after slaughtering and organic tissue samples were also selected. Yak testicular tissues were immediately stored at −80 °C. All samples were collected in strict accordance with the ethical guidelines approved by the Animal Care Commission of College of Veterinary Medicine, Gansu Agriculture University, with ethical code, GSAU-AEW-2015-0008 (5 January 2015).

### 4.2. Total RNA and MicroRNA Preparation and Sequencing

Total RNA was extracted from the yak testicular samples using Trizol reagent (TinaGen, Beijing, China), following the manufacturer’s protocol. The higher quality RNA samples were selected for constructing the library. The 470–500 bp size ligation products were enriched to generate a cDNA library for transcriptome sequencing, while the 140–160 bp size ligation products were enriched to generate a cDNA library for small RNA sequencing. The RNA and microRNA libraries were constructed as described previously [7,10,40]. The libraries were used for sequencing on an Illumina HiSeq 2500 platform (Illumina, San Diego, CA, USA). Removal of poor quality sequences and trimming of adaptor sequences from the raw sequence data were carried out by Cutadapt software (v.1.6) [41]. The cover degree and distributed situation of clean reads were counted for quality control. Samples that passed RNA quality control were used for bioinformatic analysis, which was carried out with the help of Guangzhou Sagene Biotech Co., Ltd. (Guangzhou, China).

### 4.3. RNA and MicroRNAs Analysis

The obtained sequencing reads included raw reads containing adapters or low quality bases that were filtered to remove reads containing adapters, reads containing more than 10% unknown nucleotides (N) or more than 50% low quality reads (*Q*-value ≤ 20). The remaining reads of transcriptome and tags of small RNA were further used in assembly and analysis of transcriptome and small RNA. The subsequent standard analyses were performed as described [42,43,44,45,46] with some modifications. The gene expression level of transcriptome was normalized using the FPKM method [47]. Therefore, the calculated gene expression was used directly for comparing the differences in gene expression among samples.

The microRNA sequences of some species, such as yaks, were not included in the miRBase database. For those species, the microRNA alignment with other homologous species was a dependable way to identify the known microRNAs. All of the unannotated tags were aligned with the cattle genome in this study. The microRNA expression level was calculated and normalized using transcripts per million (TPM). The candidate target genes were predicted using three software packages, including MIREAP, Miranda, and TargetScan based on the sequences of the existing microRNAs, known microRNAs, and novel microRNAs, which were more credible for use as predicted microRNA target genes.

### 4.4. Functional Enrichment and Cluster Analysis

The edgeR package [48] was applied to identify differentially expressed genes (DEGs) across samples or groups. We identified genes with a fold change ≥2 and a false discovery rate (FDR) < 0.05 in a comparison as significantly DEGs. DEGs were used for enrichment analysis of GO functions [49] and Kyoto Encyclopedia of Genes and Genomes (KEGG) pathway analysis [50]. Co-expression and interaction predictions were analyzed as described previously [9,10,23,51]. We calculated the Spearman correlation [52] for candidate DEGs to construct the correlation coefficient matrix for further investigation and determine the significance co-expressed messenger RNAs and microRNAs.

### 4.5. PCR Assays for Target Genes and MicroRNAs

The yak testis samples from different stages of maturity were utilized to confirm the different target genes and microRNAs. A poly(T) real-time PCR adaptor was specifically designed for quantifying microRNAs, as described previously [53]. The quantitative real-time PCR and semiquantitative PCR assays were used for validating DEGs as we have previously described [54,55,56] using an ABI7300 system (Applied Biosystems, Foster City, CA, USA). The experimental process and the data analysis were carried out as previously described [54]. All reactions were performed in triplicate and included controls without template.

### 4.6. Western Blot, Immunohistochemistry and Hematoxylin-Eosin Staining Assays

Western blot analyses of the testis samples for the detection of HSPA2, TDRD5, PIWIL1, RNF17, AURKA, and STAG3 were performed as described previously [54]. In brief, total protein was extracted from 100 mg of frozen tissue using RAPI (Solarbio, Beijing, China). Proteins (50 μg) were separated via 15% SDS-PAGE gel for Western blot analysis. Immunohistochemistry staining for target proteins (AURKA and STAG3, Bioss, Beijing, China) used a standard avidin-biotin-peroxidase complex method of the ABC staining system, (SABC, BOSTER, Wuhan, China). The yak testis samples were fixed in 4% paraformaldehyde. The staining of Hematoxylin–Eosin (H&E) and Immunohistochemistry was carried out as described [57,58].

### 4.7. Luciferase Reporter Assays for Key MicroRNAs and Target Genes

To detect the interactions between target genes and microRNAs, a dual luciferase reporter assay was performed as previously described [51], with some modifications. For plasmid construction, the yak messenger RNA sequences of AURKA and STAG3 were retrieved from the GenBank database. The 3′-UTR sequences of target genes were amplified from yak genomic DNA for the wild-type construct. The mutant 3′-UTR sequences of target genes were synthesized and inserted into the psiCHECK-2 vector. For the mutated-type construct, yak miR-574, miR-21-3p, and miR-2350-5p sequences were synthesized by Ubiolab Genetics Technology Company (Beijing, China). The mice Sertoli cells (TM4) were transfected using Lipofectamine™ 2000 (Invitrogen, Carlsbad, CA, USA) according to the manufacturer’s instructions. After 24 h, luciferase reporter assays were performed following the manufacturer’s instructions. This process was performed in triplicate for each target vector.

## Supplementary Materials

Supplementary materials can be found at http://www.mdpi.com/1422-0067/19/10/3083/s1.

## Abbreviations

DER	Differentially Expressed microRNA
DEG	Differentially Expressed gene
FPKM	Fragments Per Kilobase of transcript per Million mapped reads
TPM	Transcripts Per Million
GO	Gene Ontology
KEGG	Kyoto Encyclopedia of Genes and Genomes
PCR	Polymerase Chain Reaction
UTR	Untranslated Regions
