# Supplementary material for: Comprehensive Analysis of MicroRNA–Messenger RNA from White Yak Testis Reveals the Differentially Expressed Molecules Involved in Development and Reproduction
Source: Int J Mol Sci. 2018 Oct 9;19(10):3083. doi: 10.3390/ijms19103083 (PMC6213350; doi:10.3390/ijms19103083)
Supplement: Supplementary file 1 [file ijms-19-03083-s001.zip › Table S3.docx]

**Supplemental files：**

**Table S3 summary of reads annotation in small RNA sequencing**

| **Samples** | **No. total reads**  **(bp)** | **No. unique reads**  **(bp)** | **No. know**  **(%)** | **No. unknown**  **(%)** |
| --- | --- | --- | --- | --- |
| **W1-1** | 10761968 | 757571 | 8494877 | 1495244 |
| **W1-2** | 10609790 | 383104 | 8698511 | 370524 |
| **W2-1** | 10851647 | 1226473 | 8681801 | 9982242 |
| **W2-2** | 10796435 | 852959 | 8854003 | 1784190 |
| **W4-1** | 10615429 | 872106 | 8118579 | 5073560 |
| **W4-2** | 10400366 | 808325 | 885501 | 5110732 |
