# Supplementary material for: Comprehensive Analysis of MicroRNA–Messenger RNA from White Yak Testis Reveals the Differentially Expressed Molecules Involved in Development and Reproduction
Source: Int J Mol Sci. 2018 Oct 9;19(10):3083. doi: 10.3390/ijms19103083 (PMC6213350; doi:10.3390/ijms19103083)
Supplement: Supplementary file 1 [file ijms-19-03083-s001.zip › Supplemental files 1.docx]

**Supplemental files 1：**

**Table S1 Summary of detail reads in transcriptome sequencing**

| **Samples** | **Clean Data**  **(bp)** | **HQ Clean Data**  **(bp)** | **HQ Clean Data / Clean Data**  **(%)** |
| --- | --- | --- | --- |
| **W1-1** | 5627977500 | 5570003517 | 98.97% |
| **W1-2** | 5279270000 | 5027504652 | 95.23% |
| **W2-1** | 4972512750 | 4933535932 | 99.22% |
| **W2-2** | 5659318750 | 5604884716 | 99.04% |
| **W4-1** | 5051089000 | 5006853910 | 99.12% |
| **W4-2** | 4873776750 | 4829546659 | 99.09% |

**Table S3 summary of reads annotation in small RNA sequencing**

**Fig. S1 GO analysis of DEGs in transcriptome by Interproscan software**

Total of 25, 12 and 30 significantly different GO terms (*P<0.05* and *Q<0.05*) were participated in biological process, cellular component and molecular function, respectively.

| **Samples** | **No. total reads**  **(bp)** | **No. unique reads**  **(bp)** | **No. know**  **(%)** | **No. unknown**  **(%)** |
| --- | --- | --- | --- | --- |
| **W1-1** | 10761968 | 757571 | 8494877 | 1495244 |
| **W1-2** | 10609790 | 383104 | 8698511 | 370524 |
| **W2-1** | 10851647 | 1226473 | 8681801 | 9982242 |
| **W2-2** | 10796435 | 852959 | 8854003 | 1784190 |
| **W4-1** | 10615429 | 872106 | 8118579 | 5073560 |
| **W4-2** | 10400366 | 808325 | 885501 | 5110732 |


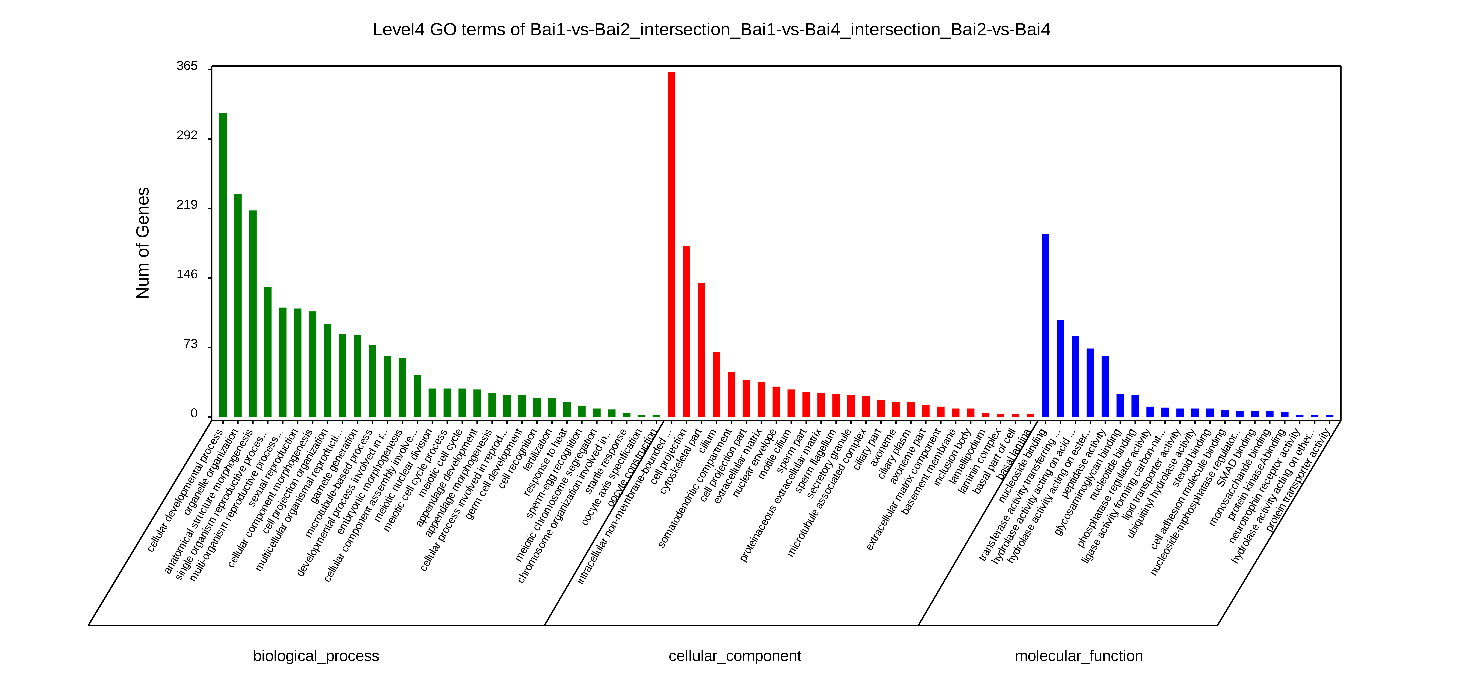


**Table S5 DEGs were predicted possible phenotypes or function by MGI database**

| Phenotypes  description | **Gene**  **No.** | Gene Symbol |
| --- | --- | --- |
| No  Phenotypes Annotation | 27 | SOX30; SPACA3; SPAG6; SPATA16; SPATA19; GLIPR1L1; FANCF; CABS1; CCDC63; CLASP1; DPCD; FSCN3; HSPA1L; IFT81; ODF4; PXT1; RRP7A; SPATA9; SPIRE2; TSGA10 ; TXNRD3; LOC102264963; LOC102273836; LOC102278413; LOC102285016; LOC102286234; LOC102287886; |
| Aberrant Spermatogenesis | 11 | IQCF1; ZPBP2; TBPL1; HOOK1; MNS1; TDRD5; ODF1; TDRD9; [TTLL5](http://www.informatics.jax.org/marker/MGI:2443657); CAPZA3; ADAD1; |
|  | 11 | AGFG1; [PIWIL1](http://www.informatics.jax.org/marker/MGI:1928897); BRDT; SPEF2; RNF17; TEX14; YBX2; SPEM1; ACR; KIAA1524; PRM3; |
|  | 4 | ATP8B3; CREM; PRSS37 ; STAG3; |
|  | 10 | HORMAD1; HSPA2; SPATA22; [GTSF1](http://www.informatics.jax.org/marker/MGI:1921424); MEIOB; RSPH1; SPDYA; SYCE3; SYCE1; TEX15; |
| Abnormal  embryo development | 14 | ARNT; AURKA; CDC20; ESPL1; FGFR1; FN1; GAB1; HNF4A; KRT8; LAMA1; ODF2; VEGFA; RXRA; BAG6; |
| Abnormal  Individual phenotypic | 15 | CCNB2; LAMA4; ITGB5; LAMB1; NAGLU; NR2F2; OSR2; PBX1; PLK4; RARRES2; SPRY2; TMEM107; TWIST1; MBNL1; ZBTB16; |
